# American Dental Association

**Published:** 1884-09

**Authors:** 


### American Dental Association.

The 20th Annual meeting of the American Dental Association was held at Saratoga, August 5th to the 8th. The meeting was well attended, especially from the Middle and Eastern States, a larger number being present than usual. The meeting was an interesting one, much good work being done. Quite a number of excellent papers were read, and for the most part were quite fully discussed. A synopsis of the papers read, together with the discussions upon them, will be presented in the October number of the REGISTER. A constant effort should be put forth on the part of the profession to make this body represent the whole profession of the country. Its work and method of procedure should be such as to embrace all.

